# Early COPD Risk Decision for Adults Aged From 40 to 79 Years Based on Lung Radiomics Features

**DOI:** 10.3389/fmed.2022.845286

**Published:** 2022-04-21

**Authors:** Yingjian Yang, Wei Li, Yingwei Guo, Yang Liu, Qiang Li, Kai Yang, Shicong Wang, Nanrong Zeng, Wenxin Duan, Ziran Chen, Huai Chen, Xian Li, Wei Zhao, Rongchang Chen, Yan Kang

**Affiliations:** ^1^College of Medicine and Biological Information Engineering, Northeastern University, Shenyang, China; ^2^Medical Device Innovation Center, Shenzhen Technology University, Shenzhen, China; ^3^Shenzhen Institute of Respiratory Diseases, Shenzhen People's Hospital, Shenzhen, China; ^4^Department of Radiology, The First Affiliated Hospital of Guangzhou Medical University, Guangzhou, China; ^5^Medical Engineering, Liaoning Provincial Crops Hospital of Chinese People's Armed Police Forces, Shenyang, China; ^6^The Second Clinical Medical College, Jinan University, Guangzhou, China; ^7^The First Affiliated Hospital, Southern University of Science and Technology, Shenzhen, China; ^8^Engineering Research Centre of Medical Imaging and Intelligent Analysis, Ministry of Education, Shenyang, China

**Keywords:** COPD risk, aging, COPD stage (GOLD), radiomics, early decision, survival Cox model, Lasso

## Abstract

**Background:**

Chronic obstructive pulmonary disease (COPD), a preventable lung disease, has the highest prevalence in the elderly and deserves special consideration regarding earlier warnings in this fragile population. The impact of age on COPD is well known, but the COPD risk of the aging process in the lungs remains unclear. Therefore, it is necessary to understand the COPD risk of the aging process in the lungs, providing an early COPD risk decision for adults.

**Methods:**

COPD risk is evaluated for adults to make an early COPD risk decision from the perspective of lung radiomics features. First, the subjects are divided into four groups according to the COPD stages. Their ages are divided into eight equal age intervals in each group. Second, four survival Cox models are established based on the lung radiomics features to evaluate the risk probability from COPD stage 0 to suffering COPD and COPD stages. Finally, four risk ranks are defined by equally dividing the COPD risk probability from 0 to 1. Subsequently, the COPD risk at different stages is evaluated with varying age intervals to provide an early COPD risk decision.

**Results:**

The evaluation metrics area under the curve (AUC)/C index of four survival Cox models are 0.87/0.94, 0.84/0.83, 0.94/0.89, and 0.97/0.86, respectively, showing the effectiveness of the models. The risk rank levels up every 5 years for the subjects who had suffered COPD after 60. For the subjects with COPD stage 0, the risk rank of suffering COPD stage I levels up every 5 years after the age of 65 years, and the risk rank of suffering COPD stages II and III & IV levels up every 5 years after the age of 70 years.

**Conclusion:**

Once the age is above 60 years, the patients with COPD need to take action to prevent the progress and deterioration of COPD. Once the age is above 65 years, the patients with COPD stage 0 need to take precautions against COPD.

## Introduction

Chronic obstructive pulmonary disease (COPD) is characterized by persistent airflow limitation. The gold standard for the diagnosis and evaluation of COPD is the forced expiratory volume in the first second (FEV_1_) and FEV_1_/forced vital capacity (FVC) ratio examined by pulmonary function test (PFT) (1). Previous studies on COPD mainly focus on COPD diagnosis and classification (2), COPD treatment (3, 4), COPD exacerbation prediction (5, 6), and COPD evaluation (7, 8). Many age-associated changes have been confirmed in the respiratory and pulmonary immune systems (9). Age relative risks of COPD mortality increase exponentially in China and the US (10). Age has become one of the factors of the score called Emphysema, Age, Smoking, SIZE (EMPHASIZE) in predicting the presence of clinically significant COPD and future morbidity (11). COPD has the highest prevalence in the elderly and deserves special consideration regarding treatment in this fragile population (9). However, it remains unclear the impact of age on the COPD risk.

The thoracic cavity's size decreases, limiting lung volumes and altering the muscles that aid in respiration with adults aging (9). The PFT result changes with the ages of both healthy people and COPD patients. After birth, the lung tissue will continue developing and growing to maturity. As a result, the alveoli and the small blood vessels in the lung will increase exponentially, and the lung volume will also become more extensive. The median FEV_1_ and FVC in the PFT increase with age from 6 to 18 years, which linearly change until the adolescent growth spurt at about 10 years in girls and 12 years in boys (12). The median FEV_1_/FVC, first, decreases, then increases, and finally decreases with age from 6 to 18 years (12). After the lungs mature, the respiratory function of the lungs gradually declines with aging. Previous research (13–18) shows that both the FEV_1_ and the FVC decline progressively with age increasing from 20 to 90 years of healthy lifelong non-smokers. The FVC and the FEV_1_ in patients with COPD also show a significant decrease during a follow-up period of 4 years (19). The FEV_1_ peaks between the age of 20 and 36 years and declines with aging (20). COPD prevalence is 2–3 times higher in people above the age of 60 years (21, 22). The increased burden of COPD seen in the elderly population may be due to age-associated changes in the structure and function of the lung, increasing the pathogenetic susceptibility to COPD (9). These changes, described in elderly lifelong non-smokers, are characterized by airspace dilatation resulting from loss of supporting tissue without alveolar wall destruction, similar to changes seen with COPD (9, 23). Therefore, it is necessary to evaluate COPD risk at different stages with aging for precision medicine.

Compared with PFT, computed tomography (CT) has been regarded as the most effective modality for characterizing and quantifying COPD (24), for example, quantitatively analyzing airway disease and emphysema in patients with COPD. Since the concept of radiomics was formally proposed in 2012 (25), radiomics of the chest CT images has been widely used for the chemotherapy response prediction in non-small-cell lung cancer (26) and pathology invasiveness prediction in patients with solitary pulmonary nodules (27). Recently, radiomics also has been used in COPD for survival prediction (28, 29), COPD presence prediction (30), and the COPD exacerbations (31). However, radiomics in COPD has not been extensively investigated yet. Currently, there are only potential applications of radiomics features in COPD for the diagnosis, treatment, and follow-up of COPD and future directions (32). In particular, lung radiomics features as an imaging biomarker that reflects the state of lung parenchyma should be applied to COPD risk evaluation for an early COPD risk decision.

In summary, our contributions in this study are briefly described as follows:

- Four survival Cox models are established to evaluate the COPD risk at different COPD stages based on lung radiomics features;
- Earlier COPD risk decisions are made. The start age of the COPD risk rank, which levels up every 5 years, is given for the subjects who had suffered COPD or may suffer COPD at different stages.

## Materials and Methods

This section mainly introduces the cohort (materials) and research methods used in this study.

### Materials

The ethics committee had approved this study of the National Clinical Research Center of Respiratory Diseases in Guangzhou Medical University, China. Chinese subjects were enrolled by the China National Clinical Research Center of Respiratory Diseases from May 25, 2009, to January 11, 2011.

**Figure 1:**
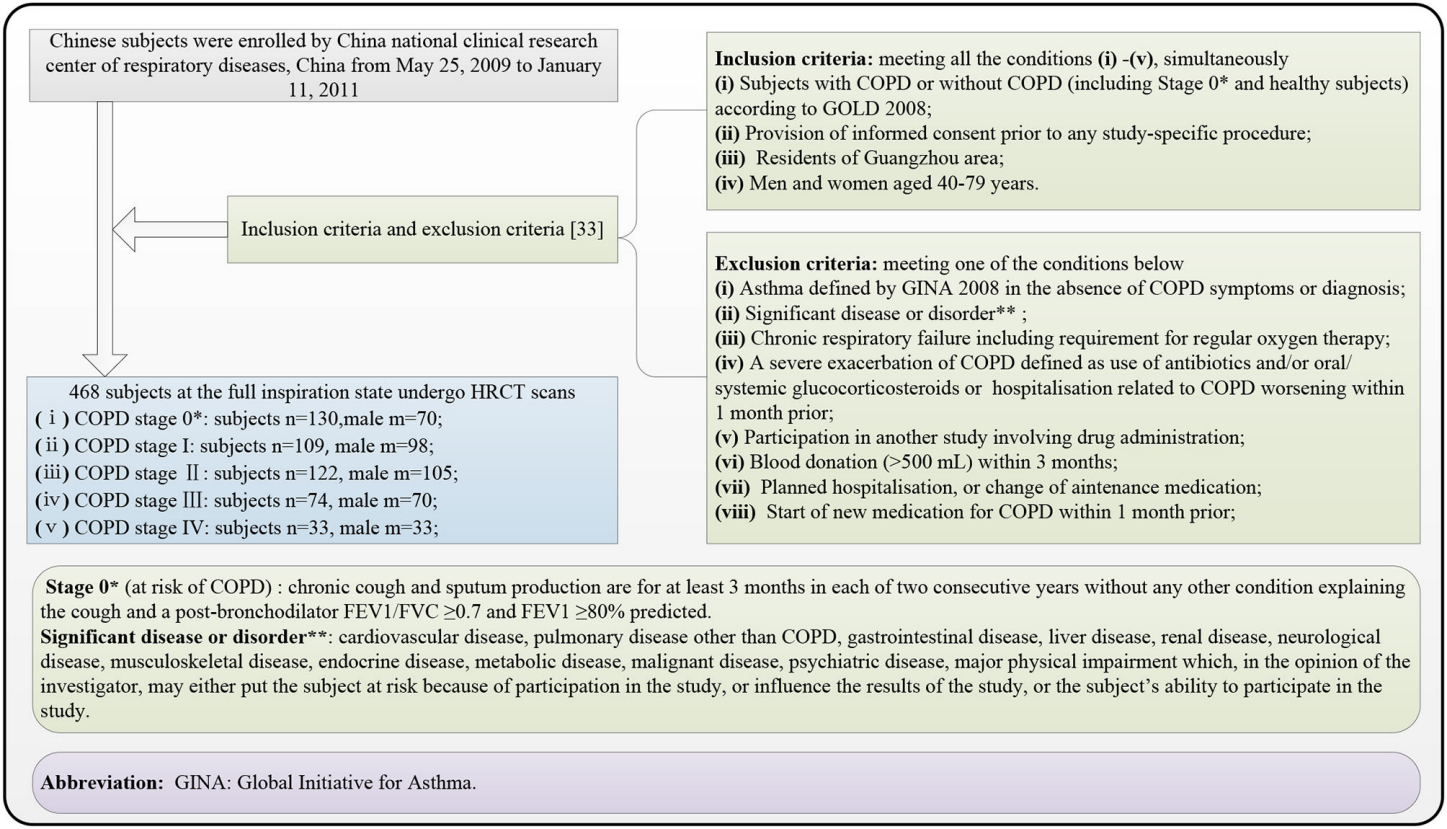
Subject selection flow diagram, finally recruiting 468 subjects suffering the chronic obstructive pulmonary disease (COPD) (stages I, II, III, and IV) and without COPD (stage 0*).

Figure 1 shows the selection flow of the subjects, followed by the inclusion and exclusion criteria (33). The 468 subjects who met the inclusion criteria and the exclusion criteria underwent HRCT scans (manufacturer: TOSHIBA, KVP: 120 kVp, X-ray tube current: 40 mA, slice thickness: 1.0 mm, window center: −600, and window width: 1,250) and PFT after using the bronchodilator. All 468 subjects had been provided written informed consent by the first affiliated hospital of Guangzhou Medical University before chest HRCT scans and PFT. The COPD stage is diagnosed from stages 0 to IV according to (Global Initiative for Chronic Obstructive Lung Disease, GOLD) 2008 criteria accepted by the American Thoracic Society and the European Respiratory Society.

### Methods

A trained deep learning model ResU-Net automatically segments the lung parenchyma images from the chest HRCT images. Then, PyRadiomics automatically calculates the lung radiomics features based on the lung parenchyma images. The 468 subjects are divided into four groups A–D according to the COPD stages, and the ages of 40–79 years are divided into eight equal age intervals in each group. Each group includes the subjects suffering from COPD stage 0 as the 1st subgroup. The 2nd subgroup of the four groups includes the subjects suffering from COPD, stages I, II, and III & IV, respectively. The Lasso model is individually applied to select the lung radiomics features of the four groups. To predict the COPD risk probability of the four groups, the four survival Cox models are constructed based on the selected lung radiomics features by the Lasso model and their age intervals, generating intuitive radiomics nomograms. The four COPD risk ranks are defined by equally dividing the COPD risk probability from 0 to 1. Subsequently, the COPD risk at different stages is evaluated with varying age intervals.

Figure 1 shows the overall block diagram of research methods in this study, including the region of interest (ROI) segmentation (refer to the “ROI segmentation” section), lung radiomics feature calculation (refer to the “Lung radiomic features calculation” section), and the COPD risk evaluation for an earlier COPD decision (refer to the “COPD risk evaluation at different stages” section).

#### Region of Interest Segmentation

**Figure 2:**
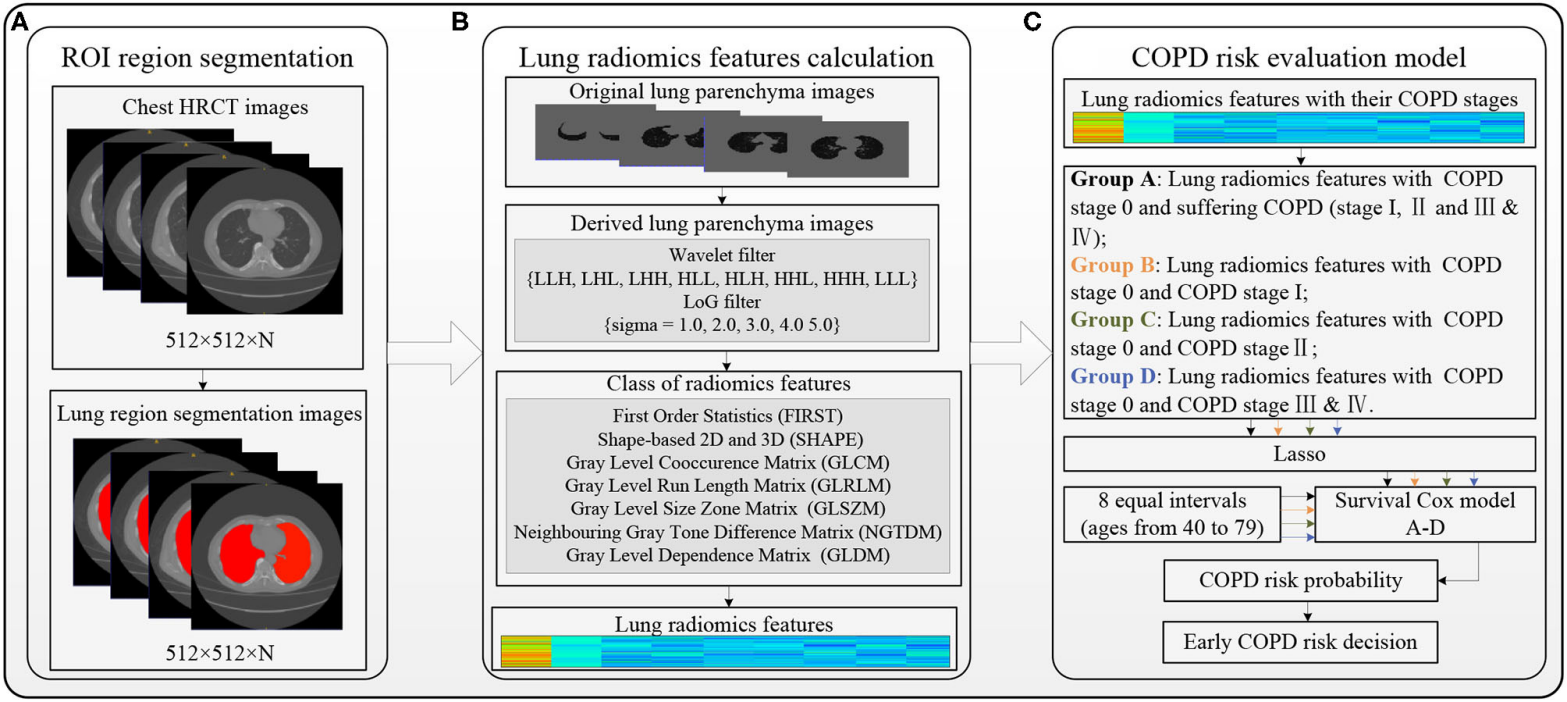
Overall block diagram of the methods in this study. **(A)** Region of interest (ROI) segmentation, **(B)** Lung radiomics feature calculation, and **(C)** COPD risk evaluation model.

Considering the overall change of lung status with aging, the lung parenchyma, including left and right lungs, is taken as the ROI in this study. The ResU-Net model trained by human chest CT images (34) automatically segment the ROI with red color in Figure 2A (size: 512 × 512 × N) from the chest HRCT images (size: 512 × 512 × N), and the detailed architecture of ResU-Net has been described in our previous study (35). All the ROI images had been checked and modified by three experienced radiologists in Shenzhen People's Hospital and the First Affiliated Hospital of Guangzhou Medical University. The trained ResU-Net model can be downloaded from the website https://github.com/JoHof/lungmask.

#### Lung Radiomics Feature Calculation

Figure 2B shows the lung radiomics feature calculation using PyRadiomics (36). PyRadiomics is available on the website https://pyradiomics.readthedocs.io/en/latest/index.html. Before calculating the lung radiomics features from the ROI (lung parenchyma) images, the ROI images with Hounsfield unit (HU) should be extracted from the chest HRCT images by our previous method (37). The original lung parenchyma images are the ROI images with HU. The wavelet filter (38, 39) and Laplacian of Gaussian (LoG) filter (40, 41) are applied to filter the original ROI images, generating two kinds of derived ROI images. The lung radiomics features are calculated based on the original and derived lung parenchyma images by the preset classes shown in Figure 2B. Finally, the 1,316 lung radiomics features for each subject are obtained.

#### COPD Risk Evaluation at Different Stages

The four groups A–D with the lung radiomics features divide according to their COPD stages, and the eight equal age intervals divide from the age 40–79 years in each group. The four groups A–D include the subjects at COPD stage 0 and suffering from COPD, COPD stages 0 and I, COPD stages 0 and II, and COPD stages 0 and III & IV, respectively. However, the advantages of optimal stability and accuracy of the least absolute shrinkage and selection operator (Lasso) model have been confirmed (42). It is applied to select the lung radiomics features from 1,316 lung radiomics features. COPD risk of each group is evaluated by the survival Cox model (43, 44) with the selected lung radiomics features and age intervals.

**Figure 3:**
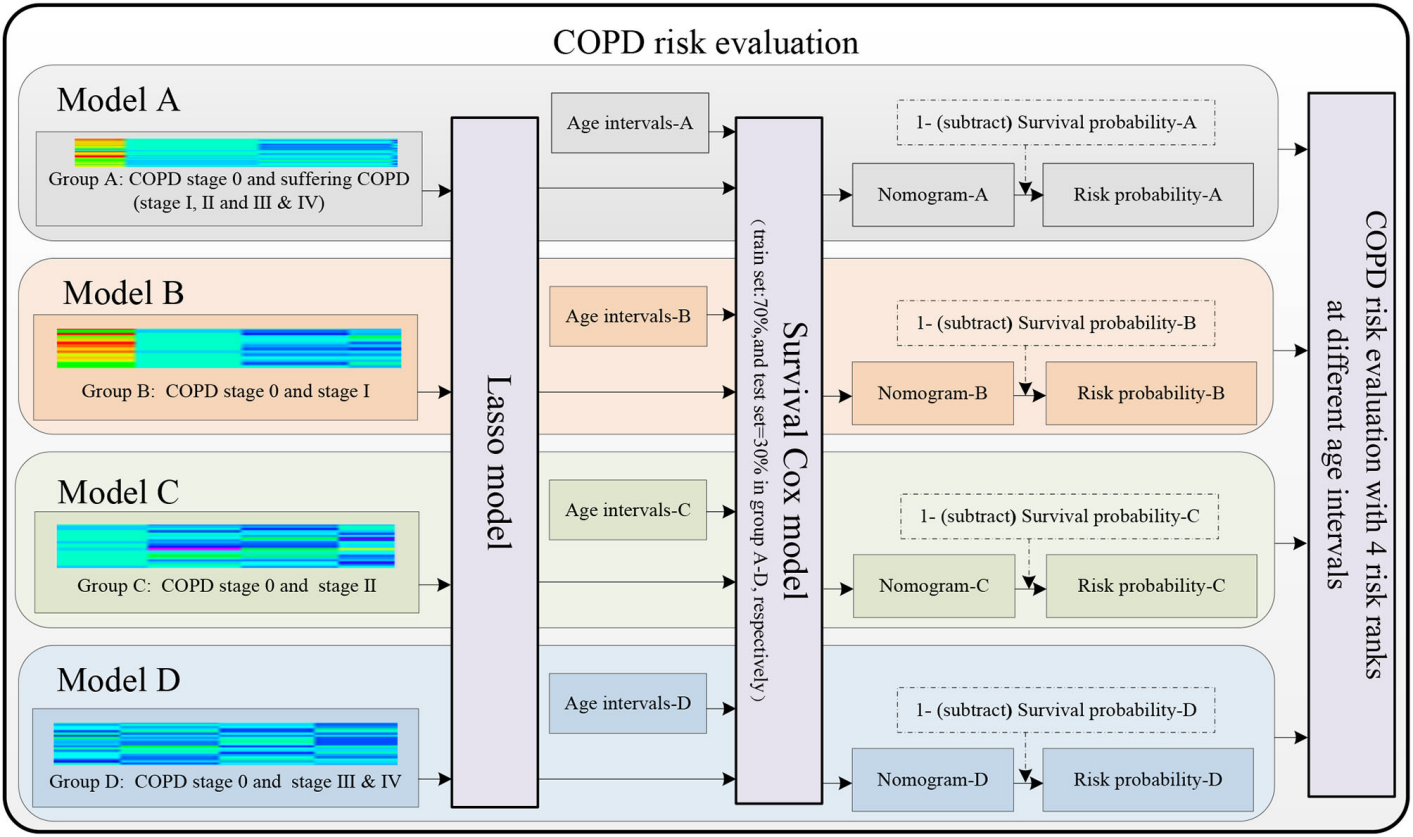
Models A–D are established to evaluate the COPD risk based on the lung radiomics features.

Figure 3 shows the four COPD risk evaluation models of the four groups A–D, including the four groups and eight age intervals (refer to the “The four groups and eight age intervals” section), the lung radiomics feature selection by Lasso model (refer to the “Lasso model for lung radiomic features selection” section), and the COPD risk evaluation by the survival Cox model (refer to the “Survival Cox model for COPD risk evaluation” section).

##### The Four Groups and Eight Age Intervals

**Figure 4:**
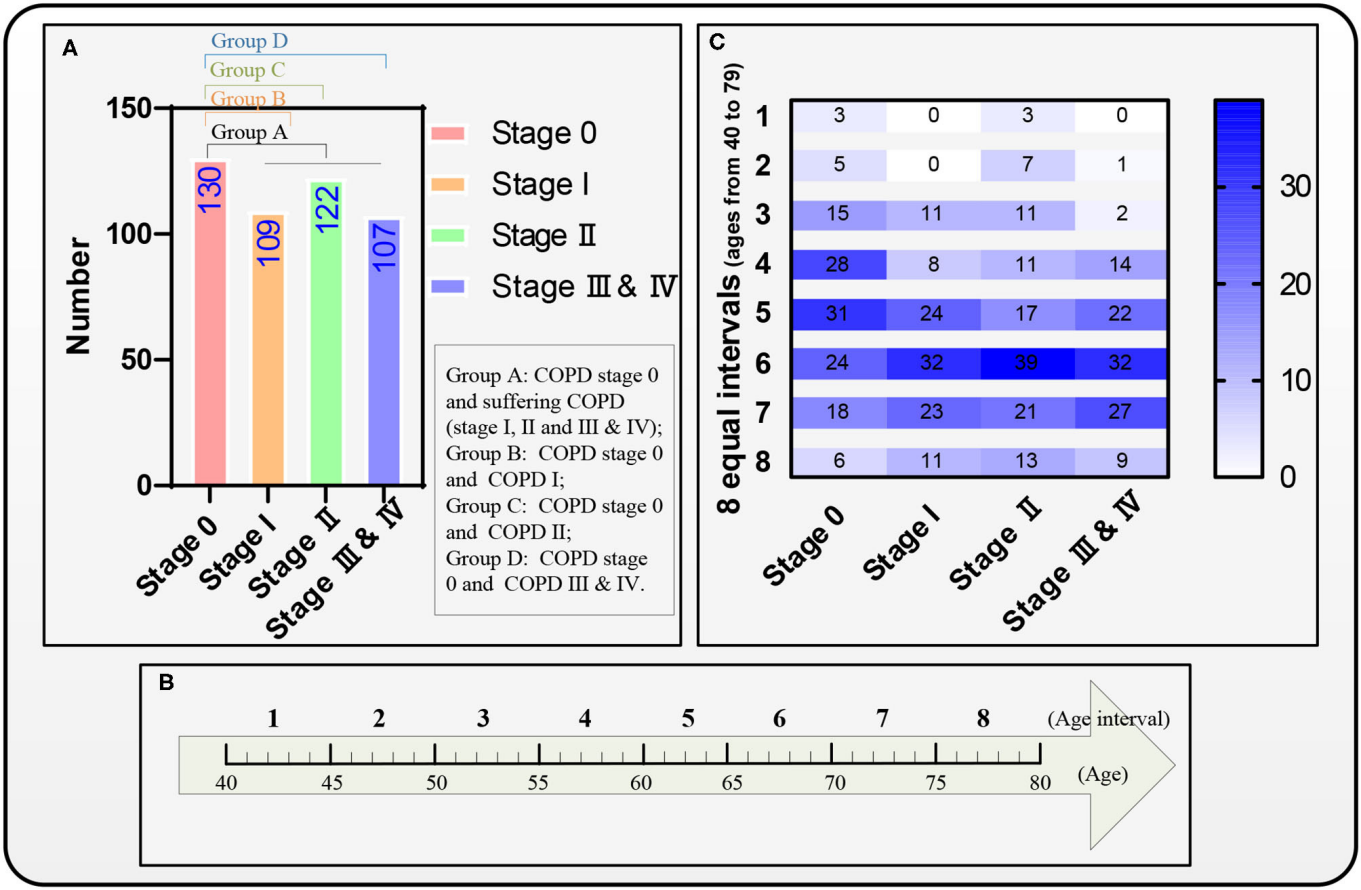
Four groups A–D, eight equal age interval divisions, and the distribution map of the eight equal age intervals. **(A)** A distribution map of the four groups A–D, **(B)** The eight equal age intervals from the age of 40 to 79 years, and **(C)** Another distribution map of the eight equal age intervals.

Four groups A–D are divided for predicting the COPD risk from COPD stage 0 to suffering from COPD (COPD stages I, II, and III & IV), stages I, II, and III & IV using survival Cox models, respectively. Figure 4A shows that all the four groups A–D include the subjects who suffer from COPD stage 0 as the 1st subgroup (*n* = 130), and the 2nd subgroup in the four groups A–D are the subjects who suffer from COPD (I, II and III & IV, *n* = 338), I (*n* = 109), II (*n* = 122), and III & IV (*n* = 107), respectively. The reference (19) has given the conclusion that the FVC and FEV_1_ in patients with COPD also show a significant decrease during a follow-up period of 4 years. Therefore, the eight equal age intervals are divided from 40 to 79 years in each group every 5 years. Figure 4B shows that the ages from 40 to 79 years of the 468 subjects are equally divided into eight age intervals. Figure 4C shows the age distribution map of eight equal age intervals at different COPD stages.

##### Lasso Model for Lung Radiomics Feature Selection

The standard R package lars (Lasso model) is applied separately to select the lung radiomics features affecting the COPD stages from the normalized lung radiomics features of the four groups. A ten-fold cross-validation (a standard R package “cv. Lars” with *K* = 10) is used to ensure the effectiveness of the Lasso model.

The lung radiomics features of the four groups are normalized by the Equation (1).


(1) $$\begin{array}{l} x_{ij}^{*}=\left(x_{ij}-\bar{x_{j}}\right)/\left(x_{jmax}-x_{jmin}\right) \end{array}$$

where, *i* = 1~468 (468 subjects), *j* = 1~1316 (1316 lung radiomics features of each subject), *x*_*ij*_ is the *i*th row and *j*th column of the 468 × 1,316 lung radiomics features, $\bar{x_{j}}$, *x*_*jmax*_, *x*_*jmin*_ are the mean, the maximum, the minimum of each radiomics feature*x*_*j*_, respectively.

Formula (2) is the Lasso model to select the normalized lung radiomics features of the four groups, respectively.


(2) $$\begin{array}{l} {\text{A}}_{k}\leftarrow arg\;min\left\{\overset{n}{\underset{i=1}{\sum }}{\left(y_{\text{i}}-\beta_{0}-\overset{p}{\underset{j=1}{\sum }}\beta_{j}x_{ij}^{*}\right)}^{2}+\text{λ}\overset{p}{\underset{j=0}{\sum }}|\beta_{j}|\right\} \end{array}$$

where matrix **A**_*k*_ denotes the selected lung radiomics features, *k* = 1, 2, 3, 4 respectively denotes group A-D. $x_{ij}^{*}$ denotes each group's normalized lung radiomics features (the independent variable). *y*_*i*_ denotes each group's COPD stage (the independent variable). Especially, *y*_*i*_ in group A denotes the COPD stage 0 and suffering COPD. λ denotes the penalty parameter (λ ≥ 0). β_*j*_ denotes the regression coefficient, *i*∈[1, *n*], and *j*∈[0, *p*].

##### Survival Cox Model for COPD Risk Evaluation

The survival Cox model (45, 46), the standard R survival package coxph, picks up the final selected lung radiomics features *x* of the four groups from the selected lung radiomics features again, by statistically significant hazard ratio (HR). Then, the subjects with the final selected lung radiomics features *x* are divided into 70 and 30%. Notably, 70% of the subjects train the survival Cox models A–D by computing estimates of the survival functions, drawing the nomograms A–D, respectively. Then, four survival Cox models adopt the standard R rms package cph with the significance level α = 0.1. Finally, 30% of the subjects in each group validate the performance of the four trained survival Cox models, respectively. It is noted that the failure event time is the age interval. The event indicator represents the COPD stage 0, and the suffering COPD, stage I, stage II, and stage III & IV of the four groups.

Equation (3) gives the survival probability formula of the survival Cox model.


(3) $$\begin{array}{l} \text{λ}\left(t|x\right)={\text{λ}}_{0}\left(t\right)▪e^{\beta^{T}x} \end{array}$$

where λ_0_(*t*) denotes the baseline hazard function, β^*T*^*x* denotes the log-risk function, which is the product of the probability at each event time the event has occurred to the individual. β denotes the weights for optimizing the Cox partial likelihood.

Equation (4) converts the COPD survival probability to the COPD risk probability.


(4) $$\begin{array}{l} Risk_{i|k}\;=\;1\;-{\text{λ}}_{i|k} \end{array}$$

where *k* = 1, 2, 3, 4 respectively denotes the group A-D, *Risk*_*i*|*k*_ denotes the *i*th COPD risk probability in the *k*th group, and λ_*i*|*k*_ is the *i*th COPD survival probability in the *k*th group as Equation (3).

Specifically, the COPD risk probability in group A is the COPD stage 0 probability and suffering COPD probability. The COPD risk probability in groups B–D is the COPD stage 0 probability and suffering COPD stage I probability, stage II probability, and stage III & IV probability, respectively. After calculating the COPD risk probability, each group's COPD risk probability is separated according to the COPD stages. Then, the four COPD risk ranks (mild risk: 0–0.25, moderate risk: 0.25–0.50, severe risk: 0.50–0.75, and extreme risk: 0.75–1) are defined by equally dividing the COPD risk probability from 0 to 1. Subsequently, the COPD risk is evaluated in the four groups at different risk ranks and age intervals.

## Results

This section shows the final selected lung radiomics features, radiomics nomograms, performance evaluation of the four survival Cox models, scatter plots, and curves of COPD risk probability of the four groups, respectively.

### The Selected Lung Radiomics Features in Groups A–D

Tables 1, 2 report the results of the selected lung radiomics features by the Lasso model and the survival Cox model's final selected lung radiomics features of the four groups, respectively. In Table 1, the symbol √ denotes the lung radiomics features selected by the Lasso model. The weight (coef), HR with 95% confidence interval (CI), and Wald's statistics (z) and significance (*p*-value) are also reported in Table 2. The symbol in Table 2 ^“***”^ denotes *p*-value <0.001, ^“**”^ denotes *p*-value <0.01, ^“*”^ denotes *p*-value <0.05, and “.” denotes *p*-value <0.1. For facilitating expression, the final selected lung radiomics features are defined as Radiomics 1–16, respectively. Radiomics 1–16 are used to construct the survival Cox models.

**Table 1:** The lung radiomics features of the four groups A–D selected by the Lasso model, respectively.

**Lung radiomics features selected by Lasso**	**Type of images**	**Class**	**Group A**	**Group B**	**Group C**	**Group D**
original_shape_Elongation	Original images	SHAPE features	√	√		√
original_shape_Maximum2DDiameterRow		SHAPE features	√	√	√	√
original_shape_Maximum2DDiameterSlice		SHAPE features			√	
original_shape_Sphericity		SHAPE features				√
original_shape_SurfaceVolumeRatio		SHAPE features	√	√		
original_firstorder_10Percentile		FIRST features				√
original_glszm_GrayLevelNonUniformityNormalized		GLSZM features			√	
original_glszm_ZoneEntropy		GLSZM features	√		√	√
log.sigma.1.0.mm.3D_firstorder_Maximum	Derived images generated from LoG filter	FIRST features			√	√
log.sigma.1.0.mm.3D_glcm_ClusterProminence		GLCM features			√	√
log.sigma.1.0.mm.3D_glrlm_GrayLevelVariance		GLRLM features			√	
log.sigma.1.0.mm.3D_glszm_SmallAreaEmphasis		GLSZM features			√	
log.sigma.1.0.mm.3D_glszm_ZoneEntropy		GLSZM features				√
log.sigma.2.0.mm.3D_firstorder_Maximum		FIRST features				√
log.sigma.2.0.mm.3D_glszm_SmallAreaLowGrayLevelEmphasis		GLSZM features			√	
log.sigma.2.0.mm.3D_ngtdm_Contrast		NGTDM features	√		√	
log.sigma.2.0.mm.3D_gldm_SmallDependenceLowGrayLevelEmphasis		GLDM features			√	
log.sigma.2.0.mm.3D_gldm_DependenceVariance		GLDM features				√
log.sigma.3.0.mm.3D_firstorder_10Percentile		FIRST features				√
log.sigma.5.0.mm.3D_firstorder_10Percentile		FIRST features	√		√	√
log.sigma.5.0.mm.3D_firstorder_TotalEnergy		FIRST features	√			
log.sigma.5.0.mm.3D_glrlm_RunLengthNonUniformity		GLRLM features		√		
log.sigma.5.0.mm.3D_glszm_SmallAreaEmphasis		GLSZM features			√	
wavelet.LLH_glcm_ClusterTendency	Derived images generated from wavelet filter	GLCM features	√			
wavelet.LLH_glszm_GrayLevelNonUniformityNormalized		GLSZM Features	√			
wavelet.LLH_glszm_LargeAreaLowGrayLevelEmphasis		GLSZM features		√		
wavelet.LLH_glrlm_GrayLevelNonUniformityNormalized		GLRLM features		√		
wavelet.LLH_firstorder_Mean		FIRST features			√	
wavelet.LLH_firstorder_RootMeanSquared		FIRST features			√	
wavelet.LHL_gldm_SmallDependenceLowGrayLevelEmphasis		GLDM features	√			
wavelet.LHL_firstorder_Kurtosis		FIRST features		√	√	√
wavelet.HLH_glrlm_ShortRunLowGrayLevelEmphasis		GLRLM features				√
wavelet.LLL_firstorder_10Percentile		FIRST features	√	√	√	
wavelet.LLL_firstorder_Minimum		FIRST features	√	√		
wavelet.LLL_firstorder_TotalEnergy		FIRST features		√		
wavelet.LLL_glcm_Imc2		GLCM features	√		√	√

**Table 2:** The final lung radiomics features of the four groups A–D selected from the survival Cox model, respectively.

**Group**	**Definition**	**Lung radiomics features selected by survival Cox**	**Coef**	**HR:exp(coef)/95%CI**	**Se(coef)**	**z**	***p*-value**
Group A	Radiomics 1	original_shape_SurfaceVolumeRatio	−0.396	0.673/ 0.531–0.853	0.121	−3.275	**
	Radiomics 2	log.sigma.5.0.mm.3D_firstorder_TotalEnergy	0.316	1.372/1.056–1.783	0.134	2.369	*
	Radiomics 3	wavelet.LLL_firstorder_Minimum	−0.248	0.780/ 0.684–0.890	0.067	−3.693	***
Group B	Radiomics 4	wavelet.LLH_glszm_LargeAreaLowGrayLevelEmphasis	0.414	1.512/1.137–2.012	0.146	2.838	**
	Radiomics 5	wavelet.LLL_firstorder_Minimum	−0.384	0.681/0.567–0.818	0.094	−4.106	***
Group C	Radiomics 6	log.sigma.1.0.mm.3D_firstorder_Maximum	0.297	1.346/1.082–1.675	0.112	2.665	**
	Radiomics 7	log.sigma.1.0.mm.3D_glszm_SmallAreaEmphasis	−0.529	0.589/0.399–0.871	0.200	−2.651	**
	Radiomics 8	log.sigma.5.0.mm.3D_firstorder_10Percentile	−0.7350	0.479/0.336–0.685	0.182	−4.045	***
	Radiomics 9	wavelet.LLH_firstorder_RootMeanSquared	−0.230	0.794/0.640–0.985	0.110	−2.096	*
	Radiomics 10	wavelet.LHL_firstorder_Kurtosis	0.529	1.697/1.214–2.373	0.172	3.091	**
	Radiomics 11	wavelet.LLL_firstorder_10Percentile	−1.085	0.338/0.223–0.513	0.213	−5.099	***
Group D	Radiomics 12	original_shape_Maximum2DDiameterRow	−0.374	0.688/0.446–1.062	0.221	−1.689	.
	Radiomics 13	original_firstorder_10Percentile	−0.654	0.520/0.358–0.756	0.191	−3.428	***
	Radiomics 14	log.sigma.1.0.mm.3D_glcm_ClusterProminence	−0.357	0.700/0.4905–0.9978	0.181	−1.972	*
	Radiomics 15	log.sigma.1.0.mm.3D_glszm_ZoneEntropy	0.461	1.585/1.064–2.364	0.204	2.262	*
	Radiomics 16	log.sigma.2.0.mm.3D_firstorder_Maximum	0.186	1.205/1.006–1.443	0.092	2.022	*

Specifically, Radiomics 1–3 are the factors that affect the COPD risk of the patients who have suffered from COPD with aging. Radiomics 4–5, Radiomics 6–11, and Radiomics 12–16 are the factors that affect the COPD risk of the patients who had suffered the COPD stages I, II, III, and IV with aging, respectively.

### Radiomics Nomograms and Performance Evaluation of Models A–D

Radiomics nomograms are pictorial representations depicting the association between radiomics variables and the probability of suffering COPD or different COPD stage events, providing an intuitive way to interpret the survival Cox model (44).

**Figure 5:**
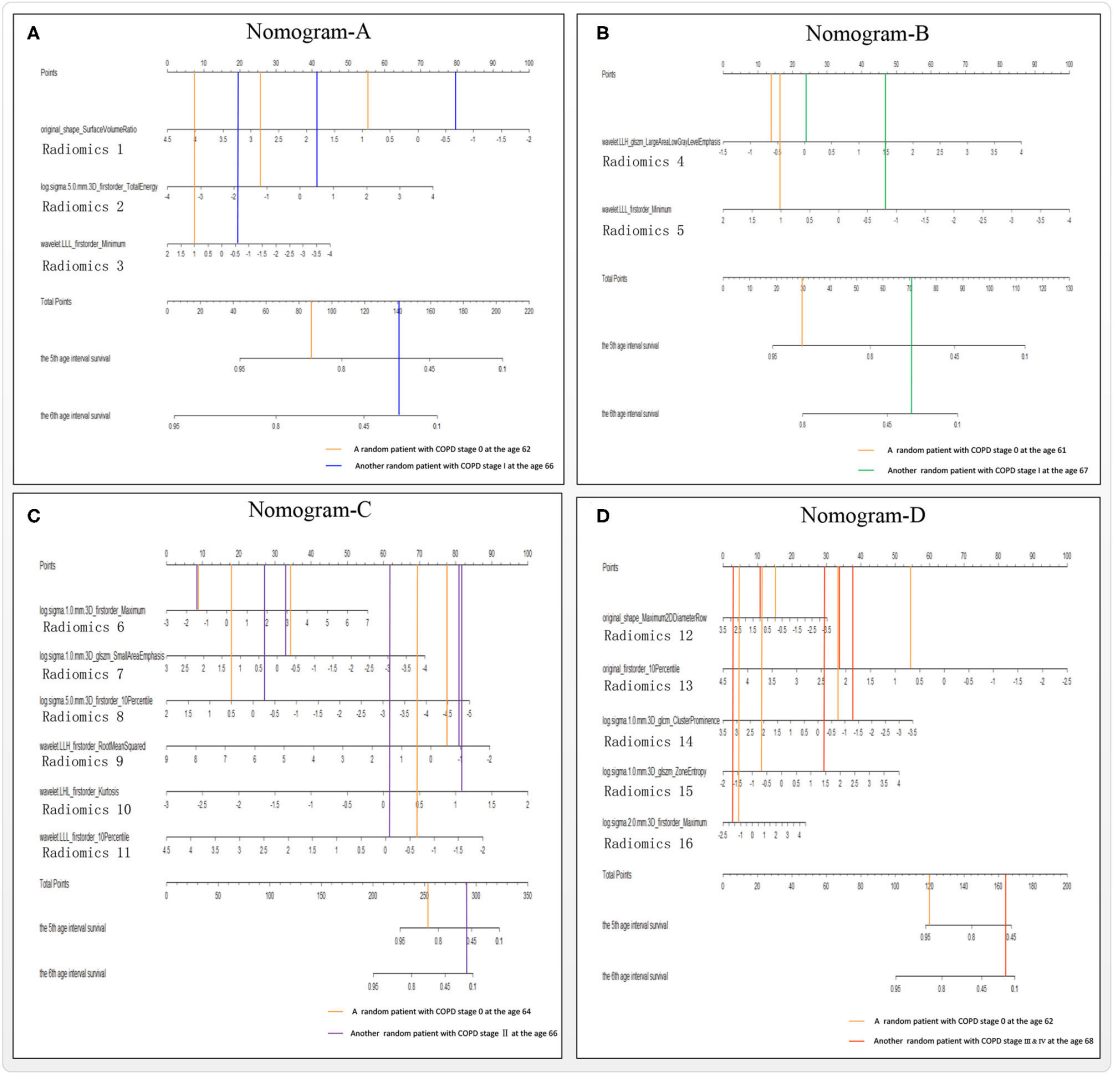
Nomograms A–D of the survival Cox models **(A–D)**, taking the 5th and 6th age interval for example.

Figure 5 shows four radiomics nomograms of the four models A–D at the 5th and 6th age interval, respectively. The radiomics nomograms of the four groups A–D further indicate the importance of the final selected lung radiomics affecting COPD with aging, respectively. The points of Radiomics *X* (*X* = 1,2, …,16) show the importance of its group. For example, Figure 5A indicates that Radiomics 2 is more critical than Radiomics 3 in nomogram-A. Figure 5B means that Radiomics 5 is more important than Radiomics 4 in nomogram-B. Figure 5C shows that the order of importance is Radiomics 10, Radiomics 9, Radiomics 11, Radiomics 8, Radiomics 7, and Radiomics 6 in nomogram-C. Figure 5D shows that the order of importance is Radiomics 13, Radiomics 14, Radiomics 15, Radiomics 12, and Radiomics 16 in nomogram-D.

**Figure 6:**
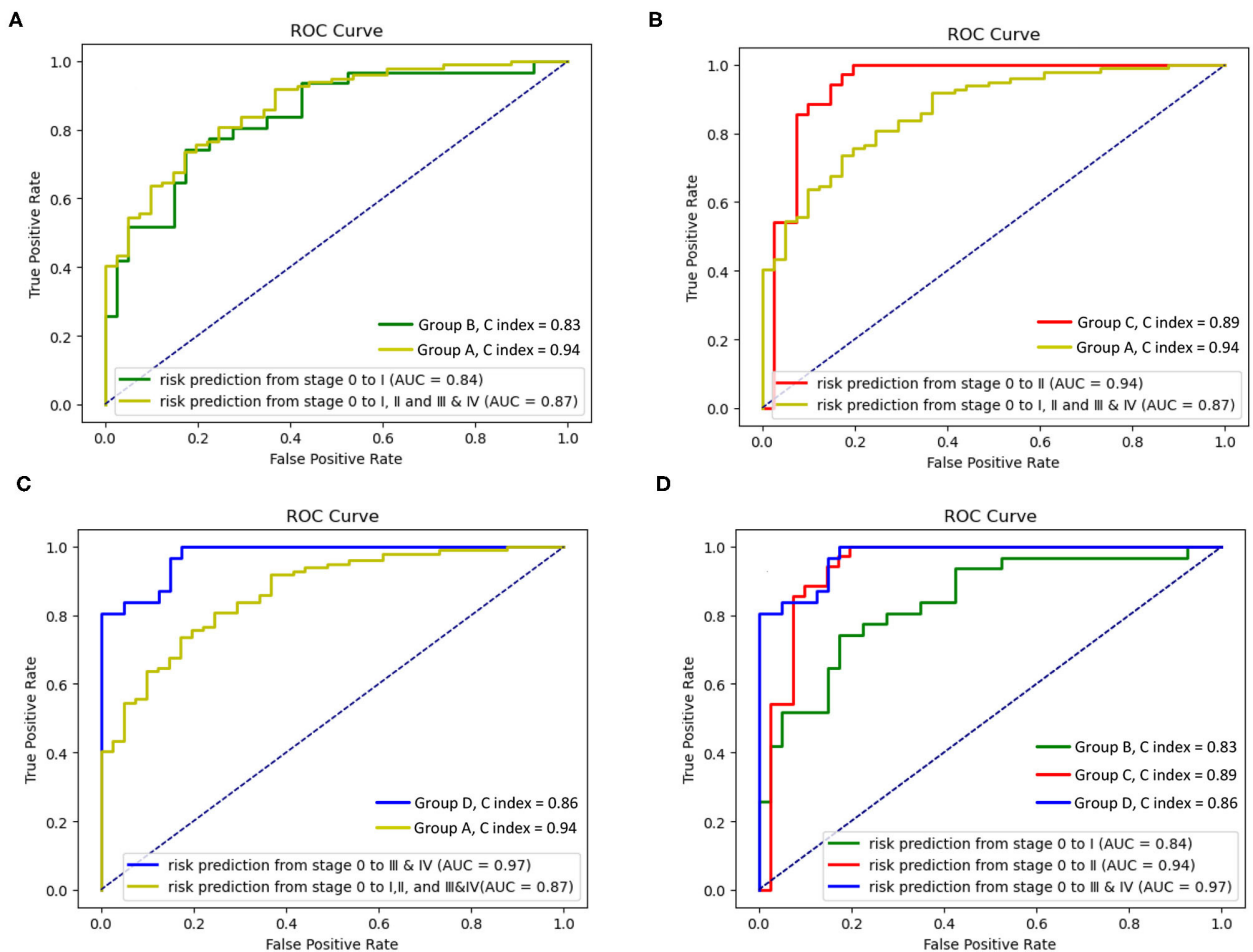
Receiver operating characteristic (ROC) curves and C index of the survival Cox model with groups A–D. **(A)** ROC curves and C index of the survival Cox model with the groups A and B, **(B)** ROC curves and C index of the survival Cox model with groups A and C, **(C)** ROC curves and C index of the survival Cox model with the groups A and D, and **(D)** ROC curves and C index of the survival Cox model with the groups B–D.

**Figure 7:**
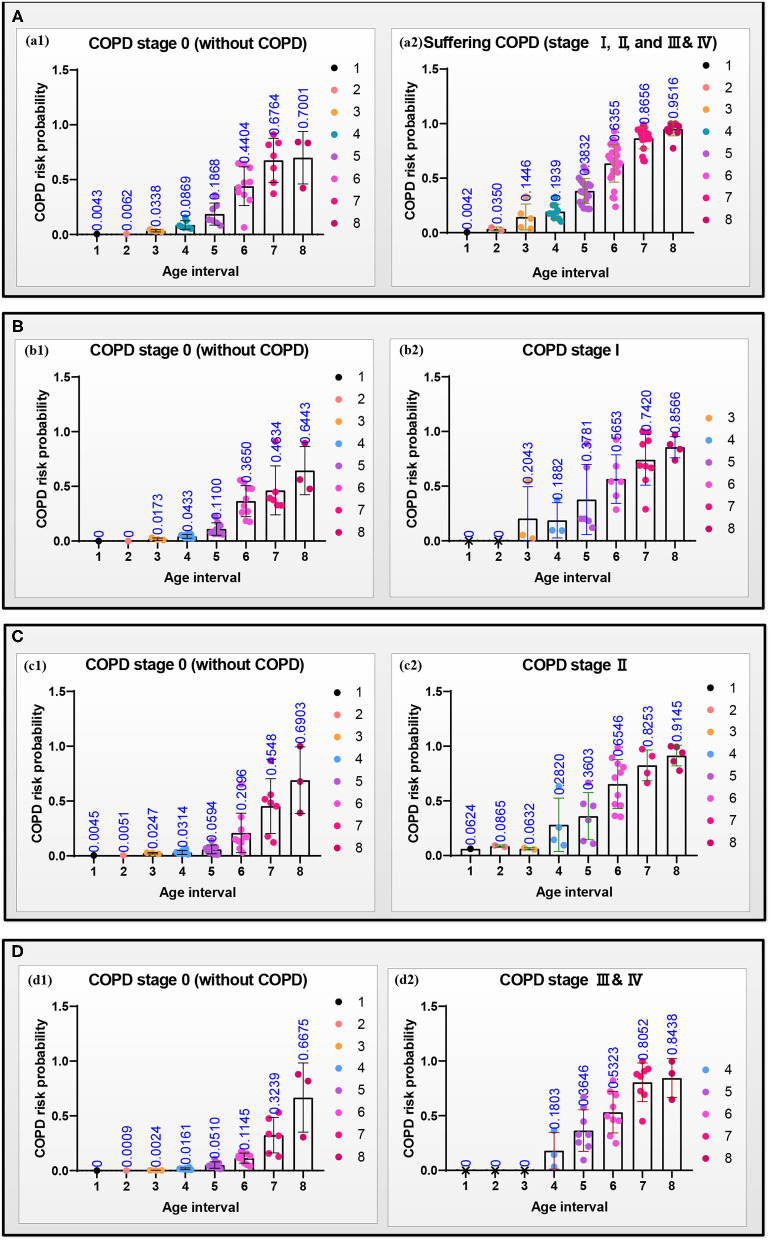
Scattering plots with bar (mean with SD) of the COPD risk probability in the four groups **(A–D)** at different age intervals, respectively. (a1–d1) The COPD risk probability of the subjects at the COPD stage 0, and (a2–d2) the COPD risk probability of the patients who had suffered the COPD, COPD stages I, II, and III & IV, respectively.

**Figure 8:**
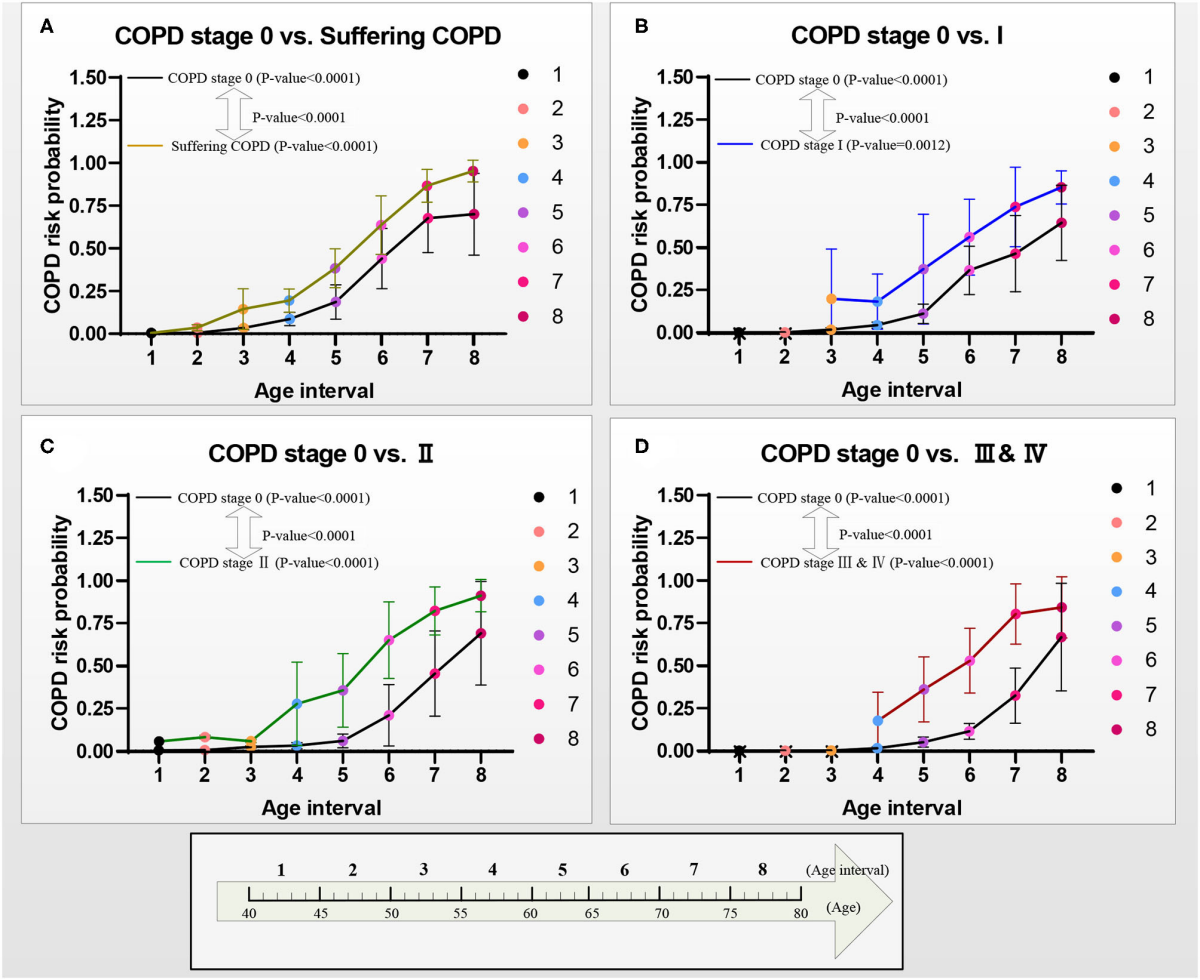
COPD risk probability curves with age increasing from COPD stages 0 to I, II, and III & IV, respectively. **(A)** The COPD risk probability in group A, **(B)** the COPD risk probability in group B, **(C)** the COPD risk probability in group C, and **(D)** the COPD risk probability in group D.

**Figure 9:**
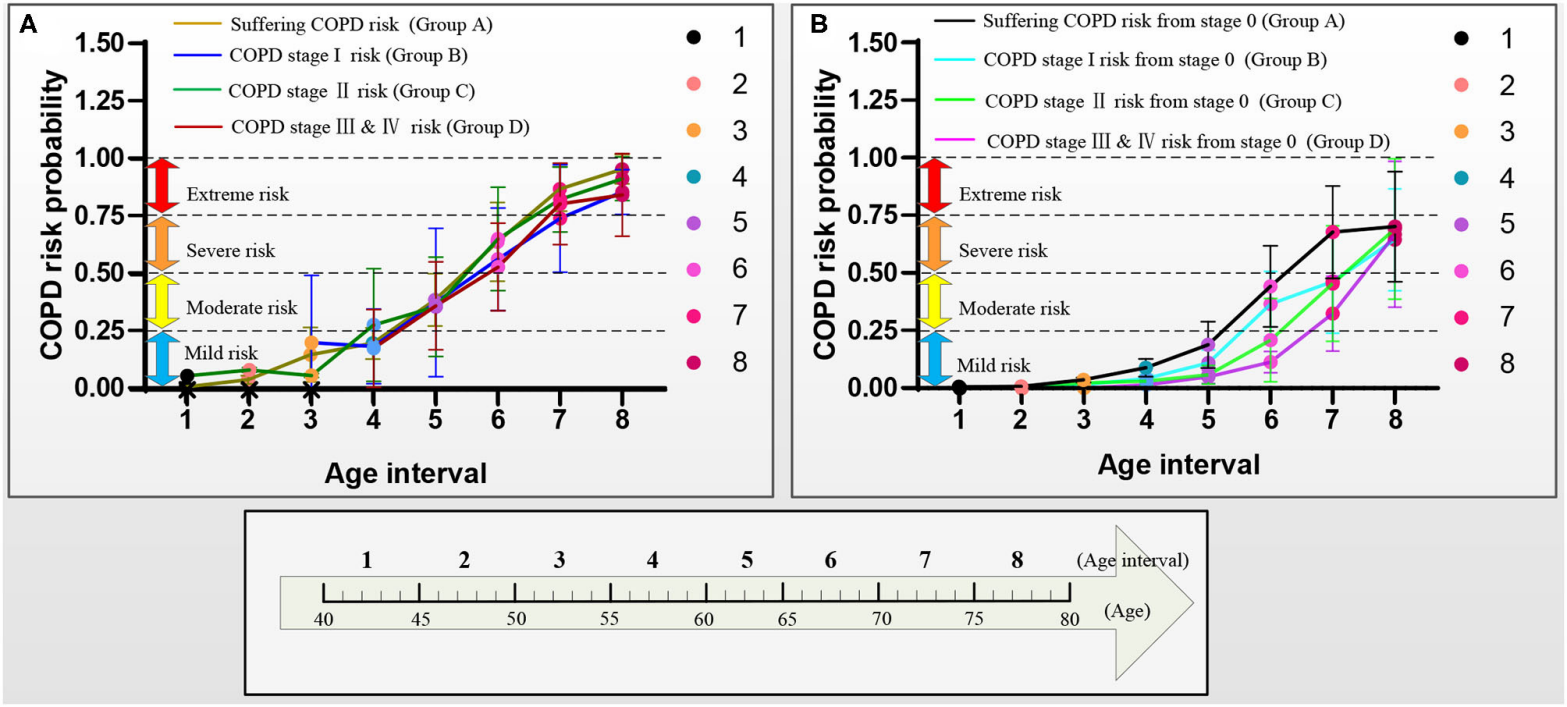
COPD risk probability curves with aging in the four groups A–D, respectively. **(A)** COPD risk probability curves of the patients who had suffered COPD, COPD stages I, II, and III & IV, and **(B)** COPD risk probability curves of subjects at COPD stage 0.

Figure 6 reports the models A–D's performances to illustrate the effectiveness of the models. After verifying the effectiveness of the models, Figure 7 intuitively reports the results of the COPD risk probability at different age intervals predicted by models A–D. Figure 8 further compares the COPD risk probability of different COPD stages at different age intervals to illustrate the impact of suffering COPD or the COPD stage on the COPD risk. Finally, Figure 9 summarizes the results in Figure 8 to make an earlier COPD risk decision for adults.

The four receiver operating characteristic (ROC) curves are drawn to evaluate the models A–D's performances. Figures 6A–C show that the area under the curve (AUC, performance measurement for classification) of model A (AUC = 0.87) is higher than that of model B (AUC = 0.84), and the AUC of the model C and the model D (model C: AUC = 0.94; model D: AUC = 0.97) is higher than that of model A (AUC = 0.87). Figure 6D shows that the AUC of the model D (model D: AUC = 0.97) is higher than that of model B and model C (model B: AUC = 0.84; model C: AUC = 0.89), and the AUC of the model C is higher than that of model B. The concordance index (C index, a standard performance metric for survival Cox analysis) of model A (C index = 0.94) is higher than other models (model B: C index = 0.83; model C: C index = 0.89; model D: C index = 0.86).

### COPD Risk Probability

Figure 7 shows the scattering plots of each group's separated COPD risk probability according to the COPD stages. Models A–D predict the COPD risk probability at different age intervals with 30% of the final selected lung radiomics features *x*. Figure 7a1–d1 show that the COPD risk probability of the COPD stage 0 in the four groups significantly increases with aging. Similarly, Figure 7a2–d2 also show that the COPD risk probability of suffering COPD, stage I, stage II, and stage III & IV in the four groups significantly increases with aging.

Figure 8 shows the curves of the COPD risk probability of the four models A–D at different age intervals. The COPD risk probability (mean with SD) of all the COPD stages 0, I, II, and III & IV in the four groups significantly increases with aging. Overall, the COPD risk probability of the patients who had suffered the COPD, COPD stages I, II, and III & IV is higher than that of subjects at COPD stage 0 in the four groups, respectively.

Figure 9 further shows the COPD risk probability curves of the patients who had suffered the COPD and the subjects at the COPD stage 0, respectively.

Figure 9A shows that the COPD risk of the patients who had suffered the COPD stages I, II, and III & IV increases with aging. Specifically, the 1st−4th age intervals of the patients who had suffered from COPD, the COPD stages I, II, and III & IV are basically at mild risk. In contrast, the 5th age interval of those patients is at moderate risk, the 6th age interval of those patients is at severe risk, and the 7th and 8 th age intervals of those patients are at extreme risk.

Figure 9B shows that the COPD risk of subjects at COPD stage 0 in the four groups increases with aging. Overall, in the same age interval, the COPD risk of the subjects at the COPD stage 0 reduces in order of suffering the COPD stages I, II, and III & IV. The risk of those subjects who may suffer from COPD is greater than that of COPD stages I, II, and III & IV with aging. Similarly, the risk of those subjects who may suffer from the COPD stage I is greater than that of the COPD stages II and III & IV with aging, and the risk of those subjects who may suffer from the COPD stage II is greater than that of the COPD stage III & IV with aging. Specifically, for the subjects with COPD stage 0, the COPD risk of the 1st−5th age intervals in groups A and B and 1st−6th age intervals in groups C and D are at mild risk. The COPD risk of the 6th age interval in group A, the 6th and 7th age intervals in group B, and the 7th age interval in groups C and D are at moderate risk. The COPD risk of the 7th and 8th age intervals in groups A and B and the 8th age interval in groups C and D are at severe risk. The COPD risk of the subjects who may suffer COPD stage I (10 years: the 6th and 7th age intervals) develops slower than that of the subjects who may suffer the COPD stages II and III & IV (5 years: the 7th age interval) in the moderate risk rank. However, until the age of 79 years, the COPD risk will not develop to the extreme risk rank.

## Discussion

In this study, four survival Cox models of the four groups A–D are developed based on the lung radiomics features to evaluate COPD risk at different stages for adults aged from 40 to 79 years, providing a COPD risk decision for adults. All four survival Cox models' effectiveness has been evaluated. They are not only effective in evaluating the risk of suffering from COPD (COPD stages I, II, and III & IV) but also effective in more detailed differentiation of COPD stages (COPD stages 0 and I, COPD stages 0 and II, COPD stages 0 and III & IV).

The lung radiomics features, as an imaging biomarker reflecting the lung structure (SHAPE radiomics features) and lung tissue (the other radiomics features except for SHAPE), are first used to evaluate COPD risk at different stages with aging. Finally, independent features Radiomics 1–16, affecting the COPD evolution from stage 0 to suffering COPD, COPD stages I, II, and III & IV, are determined. Radiomics 1, 5, 10, and 13 are the most direct risk features for COPD evolution from stage 0 to suffering COPD, COPD stages I, II, and III & IV, respectively. Our study discovers that different lung radiomics features affect the COPD risk at different stages with aging. The trends of COPD risk probability, which increase with aging, are in line with the change law of the aging process in the lung (9, 13).

For the subjects who have suffered the COPD, COPD stages I, II, and III & IV, our study discovers that although the COPD risk increases with aging, each COPD risk rank of the patients who had suffered from the COPD, COPD stages I, II, and III & IV, basically has the same age intervals. Therefore, age is not only a point to distinguish the “young COPD” (young patients with COPD aged <50 years) (47, 48) from the non-young COPD but also it can distinguish different COPD risk ranks. No matter which is COPD stage at, it is regarded as a relatively safe COPD risk rank (the mild risk) before the age of 60 years. The law of COPD risk ranks to level up with age has also been revealed for the subjects after 60 years. The risk rank of these subjects levels up every 5 years. Therefore, once the patients with COPD are above 60 years, they need to pay close attention to prevent the progress and deterioration of COPD. If necessary, manual intervention should be considered, including increasing appropriate practical exercise (49) and COPD care treatment (50) in the hospital.

For the subjects at the COPD stage 0, the COPD risk which may suffer the COPD, COPD stages I, II, and III & IV also increases with aging. Our study discovers that the age intervals of each COPD risk rank of the subjects who may suffer the COPD, COPD stages I, II, and III & IV, from the COPD stage 0 are inconsistent. Age also can distinguish different COPD risk ranks. It is a relatively safe COPD risk rank (the mild risk) of the subjects who may suffer the COPD and the COPD stage I before the age of 65 years. It is also a relatively safe COPD risk rank of subjects who may suffer the COPD stages II and III & IV before the age of 70 years. For the subjects at COPD stage 0, the risk rank of suffering COPD stage I levels up every 5 years after the age of 65 years, and the risk rank of suffering COPD stages II and III & IV levels up every 5 years after the age of 70 years. Therefore, the age of 65 years is the start age of the increased risk of the subjects who may suffer the COPD stage I, and the age of 70 years is the start age of the increased risk of the subjects who may suffer the COPD stages II and III & IV. Although the COPD risk cannot reach the extreme risk rank, once the subjects with COPD stage 0 are above 65 or 70 years, they also need to take precautions against COPD.

There are some limitations of the methods and materials in this study. First, the survival Cox model considers both events and time, but it can only analyze two opposite events. At the same time, the model itself has high requirements for the collinearity of input data. Although we used the Lasso model to remove the collinearity of the lung radiomics features, some valuable features may be omitted. Then, there is a lack of subjects aged 40–50 years in the COPD stage I and subjects aged 40–55 years in the COPD stages III & IV. That is because relatively few people aged 40–50 years who suffer from COPD stage I will go to the hospital for treatment and undergo the CT scan, and few people suffer the COPD stages III & IV at the age of 40–55 years. Finally, although lung radiomics features have met the needs of COPD risk evaluation, an improved deep learning survival Cox model (45), with a deep feed-forward neural network, has also been used to improve the model's performance further. Regrettably, the deep learning survival Cox model's AUC and C index are not enhanced. We believe that lung radiomics features with quantitative CT parameters and/or clinical text data will improve the model's performance.

## Conclusion

Four effective models are established to evaluate COPD risk from COPD stage 0 to suffered COPD, COPD stages I, II, and III & IV, respectively. The early COPD risk decision is made based on the COPD risk results. The start age of the COPD risk rank, which levels up every 5 years, is given for the subjects who had suffered COPD or may suffer COPD at different stages. It concludes that once the age is above 60 years, the patients with COPD need to be paid close attention to prevent the progress and deterioration of COPD, and once the age is above 65 years, the patients with COPD stage 0 need to take precautions against COPD.

## Data Availability Statement

The lung radiomics features supporting the conclusions of this article will be available with a reasonable request.

## Ethics Statement

The studies involving human participants were reviewed and approved by National Clinical Research Centre of Respiratory Diseases in Guangzhou Medical University, China. The patients/participants provided their written informed consent to participate in this study. Written informed consent was obtained from the individual(s) for the publication of any potentially identifiable images or data included in this article.

## Author Contributions

WL, HC, RC, and YK: conceptualization. YY, WL, YG, YL, and QL: methodology. YY, KY, SW, NZ, and WD: software. WL, YG, YL, and ZC: validation. YY, WL, HC, and XL: formal analysis. YY, WZ, and XL: investigation. KY, HC, and XL: resources. YY, WL, YG, YL, and ZC: data curation. YY: writing—original draft. WL, YL, and YK: writing—review and editing. YY, YG, QL, and SW: visualization. HC, RC, and YK: supervision. WL and YK: project administration. WL, HC, RC, and YK: funding acquisition. All authors contributed to the article and approved the submitted version.

## Funding

This work was supported by the Natural Science Foundation of Guangdong Province, China (2019A1515011382), the Stable Support Plan for Colleges and Universities in Shenzhen, China (SZWD2021010), the Scientific Research Fund of Liaoning Province, China (JL201919), and the National Natural Science Foundation of China (62071311).

## Conflict of Interest

The authors declare that the research was conducted in the absence of any commercial or financial relationships that could be construed as a potential conflict of interest.

## Publisher's Note

All claims expressed in this article are solely those of the authors and do not necessarily represent those of their affiliated organizations, or those of the publisher, the editors and the reviewers. Any product that may be evaluated in this article, or claim that may be made by its manufacturer, is not guaranteed or endorsed by the publisher.
